# FEV manoeuvre induced changes in breath VOC compositions: an unconventional view on lung function tests

**DOI:** 10.1038/srep28029

**Published:** 2016-06-17

**Authors:** Pritam Sukul, Jochen K. Schubert, Peter Oertel, Svend Kamysek, Khushman Taunk, Phillip Trefz, Wolfram Miekisch

**Affiliations:** 1 Rostock Medical Breath Research Analytics and Technologies (ROMBAT), Department of Anaesthesiology and Intensive Care, Rostock University Medical Center, Schillingallee 35, Rostock, 18057, Germany; 2 Proteomics Lab, National Centre for Cell Science (NCCS), Pune University, Pune (Maharashtra), 411007, India

## Abstract

Breath volatile organic compound (VOC) analysis can open a non-invasive window onto pathological and metabolic processes in the body. Decades of clinical breath-gas analysis have revealed that changes in exhaled VOC concentrations are important rather than disease specific biomarkers. As physiological parameters, such as respiratory rate or cardiac output, have profound effects on exhaled VOCs, here we investigated VOC exhalation under respiratory manoeuvres. Breath VOCs were monitored by means of real-time mass-spectrometry during conventional FEV manoeuvres in 50 healthy humans. Simultaneously, we measured respiratory and hemodynamic parameters noninvasively. Tidal volume and minute ventilation increased by 292 and 171% during the manoeuvre. FEV manoeuvre induced substance specific changes in VOC concentrations. pET-CO_2_ and alveolar isoprene increased by 6 and 21% during maximum exhalation. Then they decreased by 18 and 37% at forced expiration mirroring cardiac output. Acetone concentrations rose by 4.5% despite increasing minute ventilation. Blood-borne furan and dimethyl-sulphide mimicked isoprene profile. Exogenous acetonitrile, sulphides, and most aliphatic and aromatic VOCs changed minimally. Reliable breath tests must avoid forced breathing. As isoprene exhalations mirrored FEV performances, endogenous VOCs might assure quality of lung function tests. Analysis of exhaled VOC concentrations can provide additional information on physiology of respiration and gas exchange.

Breath analysis is an evolving interdisciplinary science that involves physiology and medicine with analytical chemistry and engineering. It holds promise towards noninvasive clinical diagnosis as well as therapeutic and physiological monitoring^1^,^2^,^3^,^4^,^5^,^6^,^7^,^8^,^9^,^10^. In early years, researchers were mainly focused on the discovery of trace (~ppbV to ~pptV ranges) gases in human breath as unique biomarkers for diseases. Despite the identification of more than 300 volatile organic compounds (VOCs) in exhaled breath during the last decade, not a single substance could be established as disease specific biomarker for clinical use. This was not only a consequence of inadequate fundamental knowledge on the origin, distribution and exhalation kinetics of individual markers but also due to insufficient understanding of complex physiological effects on breath VOC concentrations.

Pulmonary ventilation and perfusion are physiological determinants, which influence the alveolar gas exchange and thereby, VOC exhalation. Simple changes in physiology may have sudden and profound effects on breath VOC concentrations, which often override the actual pathophysiological effects. Recent studies have clearly demonstrated that hemodynamic changes induced by different breathing patterns or postures have immediate substance specific effects on VOC exhalation^11^,^12^. In addition, confounders such as environmental, dietary or oral/nasal cavity exposure and clinical contaminations are equally important as pathophysiological conditions or biological pathways^11^,^12^,^13^.

Recent development and application of advanced real-time mass-spectrometric (MS) techniques such as selected ion flow-tube (SIFT)-MS, proton transfer reaction (PTR)-Quadrupol-MS and PTR-Time of flight (ToF)-MS along with online end-tidal/alveolar sampling have substantially reduced several confounding variables associated with sample storage, analysis time and mixed breath matrix^14^,^15^,^16^,^17^,^18^. Such improvements in sampling, analysis and statistical validation have led to the indication that concentration changes are more important than unique breath biomarkers^11^,^12^,^13^. At present, online monitoring of instant physiological changes in exhaled VOC profiles is possible with the required analytical sensitivity or resolution, which also enables clinicians to relate different biochemical and metabolic processes to exhaled VOCs^2^,^8^,^9^,^14^,^15^,^16^,^17^,^18^.

We realized that the origin, physico-chemical characters and mostly alveolar gas exchange and compartmental distribution, are accountable for clinical interpretations of exhaled substances as breath biomarker^11^,^12^. Thus, the combined effect of the most influential physiological aspects such as hemodynamics and ventilation has to be investigated in extensive detail. We designed a study to investigate the effect of forced expiratory volume (FEV) manoeuvre onto the compositions of exhaled breath. Here, we applied real-time mass-spectrometry on healthy human subjects for breath resolved measurement of exhaled volatiles in parallel to continuous spirometry, side-stream capnometry and noninvasive monitoring of hemodynamic parameters. The following questions were addressed in detail:

- Is there any immediate physiological effect of FEV manoeuvre on VOC exhalation?
- Are those effects substance specific?
- Do such effects depend on ventilation and hemodynamic parameters?
- Can these changes be used to monitor lung functions or hemodynamic parameters?

## Results

**Figure 1:**
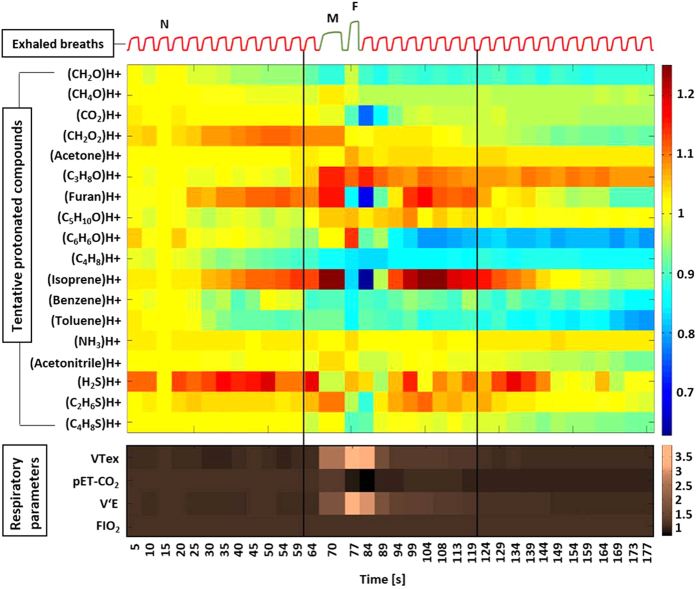
Relative changes in normalized alveolar concentrations of 18 selected compounds and respiratory parameters from 50 healthy volunteers. A schematic spirogram profile of exhaled breaths is presented on the top of the figure. Protonated compounds were tentatively identified based on their m/z ratio. VOC concentrations and respiratory parameters were normalized onto values in the third exhalation (‘N’). The beginning and the end of the second minute is represented by two vertical black lines. FEV manoeuvre was performed at the beginning of 2^nd^ minute. During the manoeuvre the maximum exhalation point is marked as ‘M’ and the forced expiration point is marked as ‘F’. VTex = expiratory tidal volume, pET-CO_2_ = end-tidal partial pressure of carbon dioxide, VE = minute ventilation and FIO_2_ = fraction of inspiratory oxygen.

Heat maps (Fig. 1) represent an overview of relative changes for selected marker substances and respiratory parameters over the course of the entire experiment. These 18 VOCs were chosen because they had significantly higher concentration in expired air than in inspired air. Profound changes of exhaled VOC concentrations were induced within seconds, when respiratory and hemodynamic parameters changed during and after the FEV manoeuvre. Inspiratory concentrations remained constant during the experiment. Detailed data on relative changes (in %), normalized mean values and variations of different VOC concentrations, respiratory and hemodynamic parameters are listed in Table 1 and Supplementary Table 1.

### Changes in exhaled VOC concentrations

**Figure 2:**
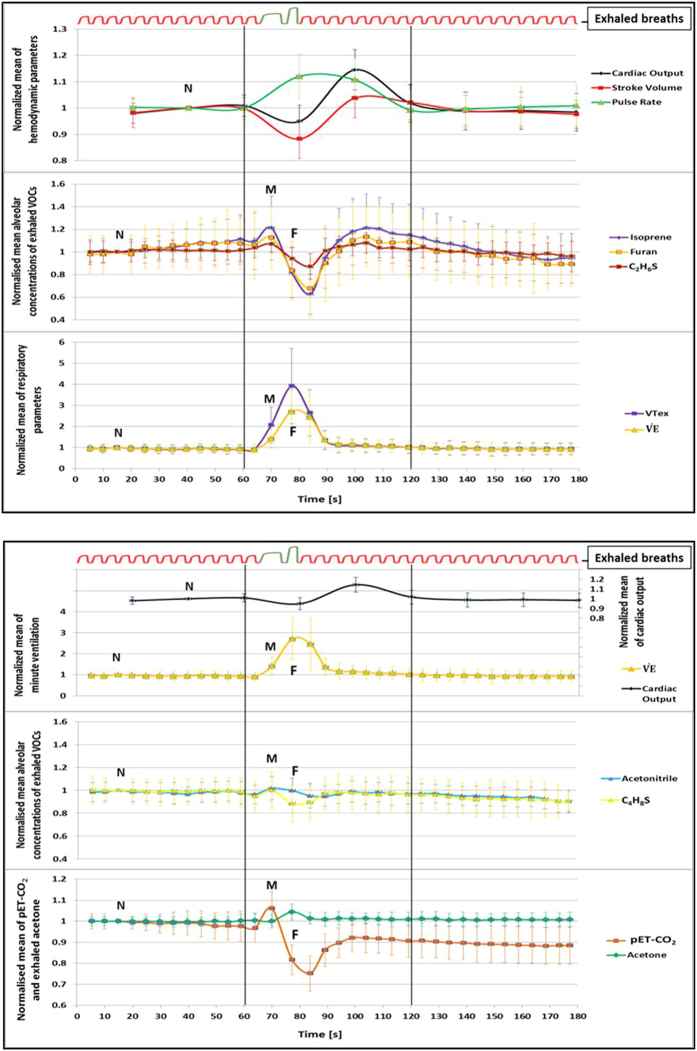
Time profiles of normalized exhaled alveolar substance concentrations, respiratory and hemodynamic parameters from 50 healthy volunteers. Diagram (**A**) represents the changes for hemodynamic [Cardiac Output, Stroke Volume and Pulse Rate] parameters (on top), VOC [Isoprene, Furan and C_2_H_6_S] concentrations (in middle) and respiratory [VTex and VE] parameters (at bottom). Diagram (**B**) shows changes for VE in primary Y axis and that of Cardiac Output in secondary Y axis (on top), VOC [Acetonitrile and C_4_H_8_S] concentrations (middle) and pET-CO_2_ and Acetone (bottom). The vertical black lines at 60^th^ and 120^th^ second represent the beginning and end of the second minute. The data points for normalization are indicated as ‘N’. “M” and “F” represent the end of Max_out_ and Forced_out_, respectively. Coloured error bars represent standard deviations. A schematic spirogram profile of exhaled breaths is presented on the top of each diagram.

All relative changes (in %) in VOC concentrations refer to the 4^th^ exhalation from the 1^st^ min (Supplementary Fig. S1). Normalized mean alveolar concentrations (normalized onto 3^rd^ exhalation) of isoprene, furan and dimethyl-sulphide (C_2_H_6_S) increased by 21%, 12% and 7%, respectively during maximum exhalation (on 70^th^ s before the forced expiration) and decreased by 18%, 16% and 6% at forced expiration (on 77^th^ s). They decreased further by 37%, 32% and 13%, respectively, just after Forced_out_ (on 84^th^ s). Concentrations of these compounds then increased again by 21%, 13% and 5% and even exceeded baseline levels (Fig. 2 A) on 113^th^ s following the time profiles of stroke volume, cardiac output and pET-CO_2_ (Table 1). Less pronounced changes were observed for concentrations of acetonitrile, allyl-methyl-sulphide (C_4_H_8_S) and isopropanol (C_3_H_8_O). In this setup, acetone concentrations increased by 4.5% at Forced_out_ (Fig. 2B). In contrast, concentrations of other compounds such as hydrogen sulphide (H_2_S), ammonia (NH_3_), formaldehyde (CH_2_O), methanol (CH_4_O), butane (C_4_H_8_), benzene (C_6_H_6_) and toluene (C_7_H_8_) etc. remained almost constant (Fig. 1).

### Changes in respiratory parameters

All relative changes (in %) in respiratory parameters refer to the 4^th^ exhalation from the 1^st^ min (Supplementary Fig. S1). Normalized mean values (normalized onto 3^rd^ exhalation) of expiratory tidal volume and minute ventilation increased by 106 and 41%, respectively during maximum exhalation (on 70^th^ s) and increased by 292 and 171% at forced expiration (on 77^th^ s). They came back to base line on 94^th^ second (Fig. 2A). In contrast, pET-CO_2_ increased by 6% at maximum exhalation, decreased by 18% at forced expiration. After that it decreased further by 25% in the next breath. From the second breath after Forced_out_, pET-CO_2_ increased again, up to 10% below the base line and then remained constant at that level (Fig. 2B). FIO_2_ remained constant throughout the experiment. V′O_2_ and V′CO_2_ were increased substantially by 29% and 88% at Forced_out_ (Fig. 1).

### Changes in hemodynamic parameters

All relative changes (in %) in hemodynamic parameters refer to the 40^th^ s time point from the 1^st^ min (Supplementary Fig. S1). Normalized mean values of stroke volume and cardiac output decreased by 14 and 6%, respectively during forced expiration, and increased by 4 and 14% in following 20 s. In contrast, pulse rate increased by 10% during FEV manoeuvre and decreased by 1% within the next 20 s (Fig. 2A). All hemodynamic values returned to base level during the first 20 s of the third minute.

Detailed statistical comparisons of differences between breaths in relation to exhaled VOC concentrations and respiratory parameters and similar comparisons for hemodynamics parameters for consecutive measurement points are shown in Table 1.

### Isoprene exhalation model for monitoring of FEV maneuvers

**Figure 3:**
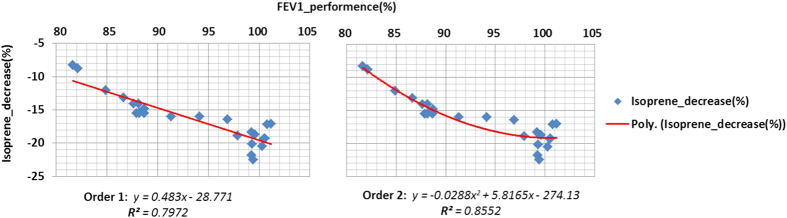
Polynomial regression models for percentages of decrease in alveolar isoprene concentration and percentages of FEV_1_ performance. The percentages of performed FEV_1_ are plotted in X-axis, Y-axis represents relative decrease in isoprene concentration. Two orders of regression equations along with the respective R2 values are presented. Isoprene decreased as the function of FEV_1_ performance, which is illustrated with a negative slope. Red lines are regressions lines of 1^st^ and 2^nd^ order.

The percentage of decrease in isoprene concentrations from Forced_out_ to following normal expiration are closely related to the percentage of FEV_1_ performances. The polynomial regression models (Fig. 3) predicting percentage of performed FEV_1_ from the decrease in isoprene concentrations are as follows:

- ***1***^***st***^***order polynomial regression equation***
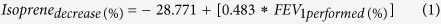
- ***2nd order polynomial regression equation***

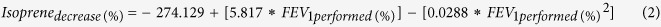


The overall results of different orders of regression are also presented in Table 2. As the third order of regression did not improve the R^2^ value significantly, it was excluded from the model.

## Discussion

In 50 volunteers we observed pronounced and immediate effects of forced respiratory maneuvers on exhaled VOC concentrations. FEV manoeuvre induced changes in both ventilation and hemodynamics, which subsequently changed VOC profiles. Correlations between concentrations of volatile substances e.g. isoprene, furan, and C_2_H_6_S and cardiac output, tidal volume, minute ventilation and pET-CO_2_ were observed. Comparatively less prominent effects were visible for C_4_H_8_S and acetonitrile. In contrast, compounds such as H_2_S, benzene, and toluene were almost unaffected by FEV manoeuvre induced physiological changes. FEV manoeuvre induced changes in VOC concentrations normalized to base line when respiratory and hemodynamic parameters went back to pre-FEV levels.

When subjects performed the FEV manoeuvre in a sitting position, they produced an extensive intrathoracic pressure, which significantly lowered venous return and ventricular preload. Eventually, according to the Frank-Starling Mechanism^19^,^20^ stroke volume and cardiac output also decreased. Due to a baroreceptor mediated increase in pulse rate^21^,^22^, cardiac output decreased moderately less than stroke volume during forced expiration. As tidal volume and minute ventilation increased during forced respiration exhaled VOCs could be expected to be diluted during these phases.

During FEV manoeuvre, all participants tried to exceed their maximal expiratory flow (MEF) rate and contracted their abdominal and intrathoracic muscles and diaphragm. As MEF depends on individual lung volume, intraluminal pressure falls rapidly from alveoli towards mouth during the effort of forced expiration. This dynamically collapsed the bronchioles, below the equal pressure point (i.e. P_intraluminal_ = P_transthoracic_) and MEF remained limited through intrathoracic airway resistance^23^. This may cause intrapulmonary shunt (lung areas, which are not ventilated but perfused)^24^,^25^,^26^.

Statistically significant differences of substance concentrations occurring within the study period can be attributed to the FEV manoeuvre induced changes in hemodynamic and respiratory parameters. CO_2_ exhalation normally depends on cardiac output, minute ventilation and the extent of dead space ventilation. As spontaneously breathing volunteers may hyperventilate due to paced breathing, pET-CO_2_ tended to decrease gradually during the last 25 s before the manoeuvre. During the FEV manoeuvre, pET-CO_2_ only partially followed the time profile that would have been predicted from stroke volume and cardiac output. This is mostly due to factors such as expiratory time, mean alveolar partial pressure of CO_2_ (PCO_2_) and alveolar slope^27^,^28^ becoming determinants for pET-CO_2_ under the conditions of forced respiration. At maximum exhalation (Max_out_) pET-CO_2_ increased significantly when stroke volume and cardiac output had already started to decrease. This increase is attributed to a change of alveolar slope during longer expiratory time, as alveolar blood-breath contact time for gas exchange was prolonged in order to empty additional lung volume (i.e. ERV). After Max_out_, pET-CO_2_ started to decrease pronouncedly during the maximum inhalation (Max_in_) and did not increase at forced expiration (Forced_out_) because of dilution during Max_in_ and due to the significant decrease in cardiac output^29^. After Forced_out_, once the participants did forced inspiration (Forced_in_) a further dilution of CO_2_ took place, which was reflected during the next normal exhalation. Although cardiac output already started to rise instantly after Forced_out_, due to the elevated pulse rate and stroke volume, pET-CO_2_ only increased from the second breath after the manoeuvre onwards. This was due to the relative hyperventilation going on until tidal volume and minute ventilation came back to baseline at the second breath after the manoeuvre. I.e. in this phase effects of cardiac output onto pCO_2_ were partially overridden by relative hyperventilation and consecutive dead space ventilation^30^,^31^.

Acetone originates from lipolysis and glycolysis^32^. In our previous study, normalized mean alveolar concentrations of acetone remained almost constant during changes in postures. No effect of hemodynamic changes was observed on acetone, mainly due to its miscibility in water, high volatility, relatively large amount of production and higher rate of distribution within lung compartments^12^. From theory, especially from multiple inert gas elimination technique (MIGET)^33^,^34^ one would expect that acetone being a highly soluble compound should behave similarly to CO_2_. However, in contrast to pCO_2_, there was a small (~4.5%) but significant (P < 0.001) increase in alveolar acetone concentrations at forced expiration. A possible explanation could be, that under the special conditions of forced expiration exchange phenomena from bronchial epithelium contributed to the exhaled concentrations of acetone^35^,^36^. Recent studies have also suggested partial exchange of VOCs with good water solubility and relatively higher blood:air partition coefficient within the bronchial tree in certain conditions such as increased bronchial perfusion or elevated exhalation flow^36^,^37^,^38^. Thus the unusual behaviour of acetone at Forced_out_ could be related to its extra-alveolar exchange within the airways. It might additionally be facilitated primarily by a pre-alveolar absorption during Max_in_ and afterwards by a post-alveolar revaporization during Forced_out_ due to the rapidly decelerating flow of forced expiration^17^.

Isoprene originates mainly from cholesterol biosynthesis^39^. Isoprene exhalation normally is positively correlated with cardiac output and negatively correlated with ventilation^12^. The increase in isoprene concentrations from first 30 s onwards is in accordance with the consecutive small increase in cardiac output and the simultaneous decrease in minute ventilation. From Max_out_ till next normal exhalation isoprene exactly followed the time profile of pET-CO_2_. Similar to CO_2_, isoprene exhalation during the FEV manoeuvre must have been influenced by factors other than cardiac output and ventilation. Due to its very low aqueous solubility (0.009 mol/L at 20 °C)^12^, alveolar concentrations of isoprene probably mirrored the pulmonary ventilation-perfusion effect during and after the manoeuvre. From the second normal exhalation after FEV manoeuvre, isoprene represented a perfusion driven increase during the following 15 s and then gradually decreased mirroring the cardiac output profile.

Other blood-borne compounds such as furan and C_2_H_6_S, which are related to smoking habits and bacterial emissions, respectively, also followed the exhalation pattern of isoprene due to their comparable solubility. Comparatively less pronounced changes were observed for compounds like acetonitrile and C_4_H_8_S, which originate from smoking/environment and dietary intakes, respectively^40^. Due to having a relatively higher aqueous solubility these two compounds did not exactly follow the behaviour of isoprene. On the other hand, H_2_S being produced mainly from oral cavity bacteria^11^,^12^, remained almost unaffected. There was a minor decrease in exhaled H_2_S concentrations during forced expiration, which could be attributed to its dilution due to higher tidal volume at that moment. Benzene and toluene are two blood borne and lipophilic compounds, which accumulate mainly from environmental exposure^40^. Due to their high rate of distribution in fatty compartments alveolar profiles of both compounds remained almost constant. Although participants were most probably not exposed to environmental compounds all in the same way, benzene, toluene, furan and acetonitrile were detectable in all 50 subjects. This is due to the variable but ubiquitous exposure to benzene and toluene and to active and passive smoking in the case of furan and acetonitrile.

Inter-individual variations are inevitable in every *in vivo* study. As our study involved only 50 volunteers, we did not try to relate any demographic parameters such as age, sex or BMI to such variations. To reduce these variations, we normalized all VOC concentrations and respiratory parameters onto their corresponding values in the third breath of the measurement cycle. Similarly, hemodynamic parameters were normalized onto values from first 40^th^ s. 10–15 breaths/min is regarded as normal breathing in healthy human adults. In order to reduce additional variations in ventilation, all participants were asked to maintain a respiratory rate of 12/min by following a metronome sound.

Execution of a correct FEV manoeuvre mainly depends on the understanding of physician’s instructions by the subject. In addition, there are several manual biases involved with both performance and analysis of such respiratory maneuvers^41^,^42^,^43^ As these pulmonary function tests are mandatory for the diagnosis of obstructive^44^ and restrictive^45^,^46^,^47^ lung conditions, it is extremely important to have a reliable and unbiased quality assurance of such test. As participants of this study did not suffer from COPD or asthma, deviations from normal FEV_1_ range and Tiffeneau Score could be attributed to low performance in doing the test. Therefore, in each participant, deviations of actual FEV_1_ and Tiffeneau score (FEV_1_/FVC ratio) from expected values (predicted from the age, gender and BMI based population studies) was rated as percentage of performance. A second order polynomial regression equation (2) turned out to reliably predict the percentage of FEV_1_ performance from the decrease in isoprene concentrations. In a perspective, exhaled concentrations of low-solubility substances such as isoprene might be used for quality control of Tiffeneau testing. This could be especially beneficial for lung function tests in sports medicine and physical fitness tests in healthy subjects and athletes.

As exhaled substance concentrations markedly depend on physiological parameters, such as breathing patterns, flow and airway resistances, unforeseeable and highly variable effects onto results have to be expected when any forced and non-physiological breathing maneuvers are used during breath sampling. Results of this study demonstrate that knowledge from pulmonary and airway gas exchange theory has to be taken into account when breath sampling is to be done in a reliable way. Analysis of exhaled VOC concentrations can provide additional information on physiology of respiration and gas exchange. Our results underline the importance of control and standardization of breath sampling with respect to tidal volume, respiratory rate, flow and airway resistance. In a perspective, breath VOCs could be used for quality assurance of lung function tests.

## Methods and Materials

All experiments were carried out in accordance with Declaration of Helsinki guidelines. All experimental protocols were approved (Approval number: A 2015-0008) by the Institutional Ethics Committee (University Medical Centre Rostock, Rostock, Germany) and signed informed consents from all subjects were obtained before conducting measurements.

### Experimental setup

**Figure 4:**
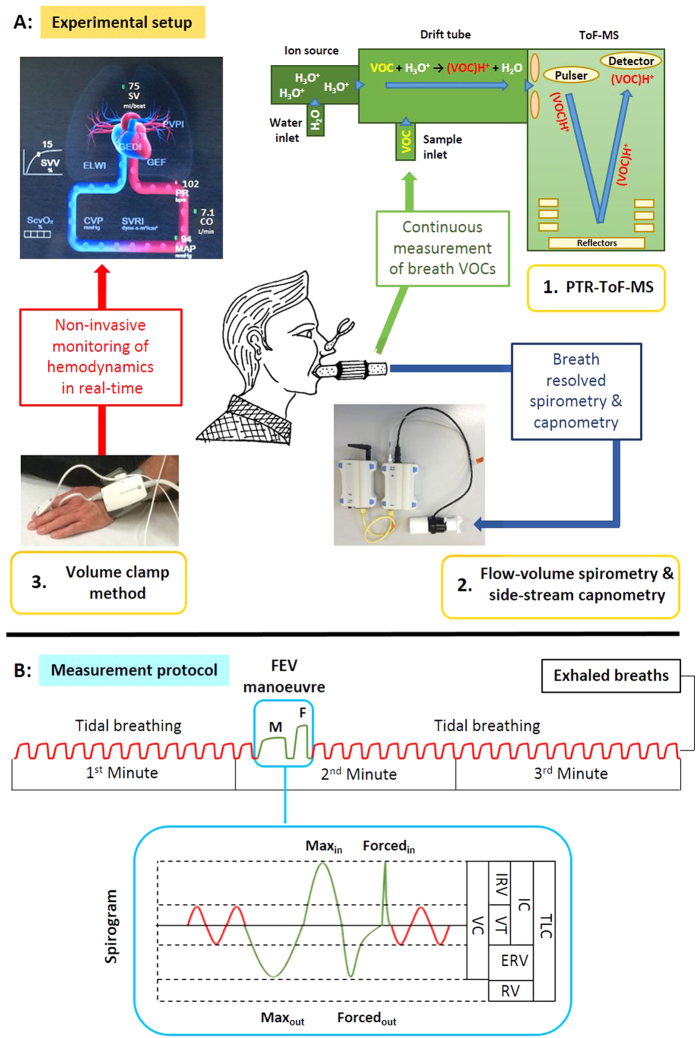
Experimental setup [**A**] and the measurement protocol [**B**] of the study. [**A**] Parallel monitoring of breath VOCs by PTR-TOF-MS (**1**), lung function parameters and pET-CO_2_ by means of spirometry and capnometry (**2**) and hemodynamic parameters such as SV = stroke volume, CO = cardiac output, PR = pulse rate and MAP = mean arterial pressure (**3**). **[B]** Schematic spirogram of the whole experiment during tidal breathing and FEV manoeuvre. Spirogram of FEV manoeuver: VC = vital capacity, VT = tidal volume, ERV = expiratory reserve volume, IRV = inspiratory reserve volume, IC = inspiratory capacity, RV = residual volume and TLC = total lung capacity.

We combined three devices, for real-time measurements of several parameters in parallel (Fig. 4A).

- *PTR-ToF-MS* for continuous monitoring (time resolution: 200 ms) of breath VOC concentrations.
- *Flow-volume spirometry and side-stream capnometry* for breath-resolved measurement of lung function (e.g. respiratory rate, tidal volume, minute ventilation) and pET-CO_2_.
- *Volume clamp method* for non-invasive determination of hemodynamics (e.g. blood pressure, cardiac output, pulse rate).

All devices were synchronised, and data acquisition was initiated simultaneously.

### Measurement protocol for healthy volunteers

Demographic data from 50 healthy participants (27 males, 23 females; aged between 21–54 years) are shown in Table 3. In order to explain and prepare the test procedures, volunteers were requested to appear at least 30 min before the actual measurements. Participants were asked to breathe normally (Respiratory rate = 12/minute) through a sterile mouthpiece following a metronome sound to determine normal tidal volume (VT). After 13 normal breaths, volunteers performed the forced expiratory volume (FEV) manoeuvre and then again breathed normally until the end of the third minute (Fig. 4B).

The FEV manoeuvre consists of 4 distinct steps:

- a maximum exhalation (Max_out_): Exhalation of tidal volume (VT) and expiratory reserve volume (ERV)
- a maximum inhalation (Max_in_): Inhalation of the vital capacity (VC) corresponding to tidal volume (VT), expired (ERV) and inspired (IRV) reserve volumes,
- followed by a forced expiration (Forced_out_) to exhale the vital capacity (VC) as fast as possible
- and finally a forced inspiration (Forced_out_) to inhale the vital capacity corresponding to tidal volume (VT), expiratory (ERV) and inspiratory (IRV) reserve volumes as fast as possible (spirogram in Fig. 4 B).

Nose clips were used to prevent any partial nasal exhalation during the measurements. According to ERS guidelines, we repeated the entire measurement cycle (3 min) in all volunteers to check any possible differences in the performance and execution of the FEV manoeuvre. As we did not find any significant differences between these measurements, the first test of each participant was used for statistical evaluation.

#### PTR-ToF-MS for breath VOCs analysis

##### Instrumentation

We used an online PTR-ToF-MS-8000 (Ionicon Analytik GmbH, Innsbruck, Austria) for continuous real-time measurement of volatile organic compounds (VOCs) in breath. The working principle and conditions of the instrument for breath sampling were described in several studies^11^,^12^. Concisely, the soft ionisation of VOCs is based on a non-dissociative proton transfer reaction [VOC + H_3_O^+^ → (VOC)H^+^ + H_2_O], which ionises VOCs with relatively higher proton affinity than water. Protonated VOCs are then detected in a high resolution reflectron time-of-flight mass spectrometer (Tofwerk AG, Thun, Switzerland) according to their mass to charge (m/q) ratio. There is no requirement for preconcentration and ambient air can be used as buffer gas. We sampled breath in a continuous side-stream mode by using a 6 m long inert and heated silco-steel transfer-line (ID 0.75 mm, Restek, Bellafonte, USA), which was connected to the sterile mouthpiece. Table 4 shows, PTR-ToF-MS-8000 measurement parameters.

A time resolution of 200 ms for data acquisition was realized by the associated ToF-DAQ Software. After every minute of measurement, a file was automatically recorded and the mass scale was calibrated by using the following masses: H_3_O^+^-isotope: 21.0226 Th; NO^+^: 29.9980 Th; protonated acetone: 59.049 Th.

##### Data processing

All VOCs were measured in counts per seconds (cps) and their intensities were normalised onto primary ion (H_3_O^+^) counts. We used a custom-made data processing algorithm called ‘breath tracker’ (MATLAB version 7.12.0.635, R2011a) to identify alveolar and inspiratory phases of breath^11^,^12^. Any endogenous and blood-borne compound that has relatively higher signal intensity in expiration can be used as a tracker mass. In our study, acetone was used as the tracker mass.

#### Spirometry and capnometry for measurements of respiratory parameters

We conducted breath-resolved spirometry and side-stream capnometry by using the Oxycon Mobile device (CareFusion GmbH, Hoechberg, Germany). Subsequent data analysis was performed by the JLAB Software 5.3x (Version 02.00). This equipment complies with all criteria of both European Respiratory Society (ERS) and American Thoracic Society (ATS) standards and has been validated for clinical and laboratory practice^48^,^49^.

The main device consists of a flow-volume sensor (TripleV unit), a gas sensor box (SBx/CPX unit), a data exchange unit (DEx transmitter) and a power calibration unit (PCa receiver). An ambient unit and a gas analyser are integrated in the SBx unit, which determine the ambient conditions and O_2_/CO_2_ content in calibration gas (Standard content: 5% CO_2_ and 16% O_2_), respectively. The TripleV unit comprises of a rotatory flow sensor, a main-stream volume sensor (electrical infrared) and a sample tube, connected to the side-stream capnometer (integrated in SBx unit). The TripleV-SBx unit allows continuous breath-by-breath and intra-breath measurements of spirometric (lung functions) and capnometric (pET-CO_2_) values in real-time. The DEx is used for recording, storage and telemetric transfer of data to the PCa unit. The PCa unit receives telemetric data as a PC-interface to the JLAB Software and is also used actively during calibrations. Prior to inclusion of every new participant, measurement of the ambient conditions as well as volume and gas calibrations were performed. General respiratory parameters such as respiratory rate, tidal volume, minute ventilation, FIO_2_ and pET-CO_2_ etc. were recorded breath-by breath. The FEV manoeuvre was performed during an intra-breath phase and additional lung functions such as FEV_1_ (forced expiratory volume in 1^st^ s), FVC (forced vital capacity) and EELV (end expiratory lung volume) etc. were recorded for subsequent time points. Additional metabolic parameters such as RER, V′O_2_, V′CO_2_ were assessed by this system. These values usually are determined during stable conditions of rest or exercise^50^,^51^. As these values are calculated from spirometric data, the sudden and exceptional manoeuvre our volunteers were told to do induced artificial variations which, therefore, do not mirror any real change of these metabolic parameters.

#### Volume clamp method for non-invasive monitoring of hemodynamics

Any invasive approach for hemodynamic monitoring in healthy volunteers is not possible for ethically reasons. Thus, we used a ClearSight System EV1000 (Edwards Lifesciences, California, USA) for continuous, non-invasive measurement of hemodynamic parameters in real-time. The instrument and associated volume clamp method are described in previous study^12^. Briefly, a suitable finger-cuff is used for each participant to clamp the middle phalanges of either index or middle finger. A heart reference sensor calibrates the relative position of the finger-cuff with respect to the heart. The finger-cuff (with infrared sensing technology) and the pressure controller measure blood pressure variations over time, which results in a photo-plethysmogram. The initial blood pressure waveform and associated parameters are recorded approximately in first 20 s. Blood pressure, pulse rate, stroke volume and cardiac output are recorded and calculated in 20 s intervals from the arterial waveform, which is derived from the photo-plethysmogram and pressure waveform. Although the standard errors for absolute values in this system range between 20% in healthy subjects, relative changes in all parameters are tracked with acceptable accuracy^12^,^52^.

### Statistical analysis

#### Comparison of differences

Every fourth breath along with the maximum and forced exhalation (from the 2^nd^ min) was included for statistical comparisons for VOC concentrations and respiratory parameters. We excluded the hemodynamic data from the first 20 s of the measurement cycle to avoid any possible artefact from the arterial waveform calibration time of the ClearSight System. Thus every data point for hemodynamic parameters was compared to the 40^th^ s time point from the first minute.

Statistically significant differences between VOC concentrations, respiratory and hemodynamic parameters were determined by means of repeated measurement ANOVA on ranks (Friedman repeated measures analysis of variance on ranks, Shapiro-Wilk test for normal distribution and post hoc Student–Newman–Keuls method for pairwise multiple comparisons between all groups; P-value < 0.05) using SigmaPlot (version 13). From all pairwise comparisons, we selected those referring to the 4^th^ breath and 40^th^ s of the 1^st^ min. For a minimum detectable difference in mean substance intensities of 400 cps and an estimated standard deviation of 400, 10 groups, each group size of 50 and alpha = 0.05 the power of the test was 0.959. A schematic overview of the statistical comparisons is presented in Supplementary Fig. S1.

#### Correlation analysis

In order to investigate correlations between the behaviour of VOC concentrations and the respiratory and hemodynamic parameters during and after the manoeuvre, we applied polynomial regression analysis (SigmaPlot; version 13).

#### Modelling of VOC exhalations to monitor FEV performances

FEV is a standard respiratory manoeuvre, which is discussed in the measurement protocol. Each manoeuvre consists of two exhalations; a maximum exhalation (Max_out_) followed by a forced expiration (Forced_out_). In order to model some VOCs of interest during and after the manoeuvre those two exhalations and the very next normal exhalation were used for modelling. As the volume of Max_out_remained very reproducible in any individual, we reduced inter-individual variations in our analysis by normalizing the absolute VOC concentrations in both Forced_out_ and the following normal exhalation onto the Max_out_ point for each participant. Forced vital capacity (FVC) is the total volume of Forced_out_ after a Max_in_ and FEV_1_ is the volume exhaled within the 1^st^ s of forced expiration. FEV_1_/FVC ratio (Tiffeneau-Pinelli index) is an important parameter in lung function testing within the field of pulmonology and respiratory medicine. Performance of FEV_1_ and analysis of Tiffeneau score lies on considerable manual biases. In order to establish an unbiased quality control of such test, we investigated the dependency of VOC exhalations on the maneuvers. Thus, we compared the relative changes in substance concentrations with the percentage of individual FEV_1_ performances (performed % of the predicted value from age, sex and BMI based population studies) by means of polynomial regression (SigmaPlot; version 13).

## Additional Information

**How to cite this article**: Sukul, P. *et al*. FEV manoeuvre induced changes in breath VOC compositions: an unconventional view on lung function tests. *Sci. Rep.* **6**, 28029; doi: 10.1038/srep28029 (2016).

## Supplementary Material

Supplementary Information

## Figures and Tables

**Table 1:** Changes in normalized concentrations of six different VOCs and changes (A,B) in normalized values of respiratory (A) and hemodynamic parameters (B).

**A**									
** Protonated VOCs Mass [gm mol**^**−1**^]	**Minute**	**Breath**	**(%)Change of normalized mean values**	**Significance/Breath (P-value)**	**Respiratory parameters**	**Minute**	**Breath**	**(%)Change of normalized mean values**	**Significance/Breath (P-value)**
Comparison point	1^st^	4^th^	0	N/A	Comparison point	1^st^	4^th^	0	N/A
(Isoprene)H^+^69.06989	1^st^	8^th^	6.08	<0.001	Expiratory tidal volume [L]	1^st^	8^th^	−8.57	0.070
	1^st^	12^th^	11.12	0.001		1^st^	12^th^	−8.03	0.175
	2^nd^	Max	21.18	<0.001		2^nd^	Max	106.28	<0.001
	2^nd^	Forced	−18.41	<0.001		2^nd^	Forced	291.66	<0.001
	2^nd^	4^th^	−37.26	<0.001		2^nd^	4^th^	164.99	<0.001
	2^nd^	8^th^	21.16	<0.001		2^nd^	8^th^	7.30	<0.001
	3^rd^	1^st^	12.33	<0.001		3^rd^	1^st^	−0.11	0.020
	3^rd^	5^th^	1.62	0.668		3^rd^	5^th^	−4.67	0.063
	3^rd^	9^th^	−5.03	<0.001		3^rd^	9^th^	−7.57	0.004
Comparison point	1^st^	4^th^	0	N/A	Comparison point	1^st^	4^th^	0	N/A
(C_2_H_6_S)H^+^ 63.0263	1^st^	8^th^	1.31	0.914	Minute ventilation [L/min]	1^st^	8^th^	−5.74	0.020
	1^st^	12^th^	1.68	0.668		1^st^	12^th^	−6.91	0.282
	2^nd^	Max	6.99	<0.001		2^nd^	Max	41.36	<0.001
	2^nd^	Forced	−5.79	<0.001		2^nd^	Forced	171.40	<0.001
	2^nd^	4^th^	−12.75	<0.001		2^nd^	4^th^	145.77	<0.001
	2^nd^	8^th^	4.96	<0.001		2^nd^	8^th^	11.16	<0.001
	3^rd^	1^st^	3.90	<0.001		3^rd^	1^st^	−0.19	0.154
	3^rd^	5^th^	−1.50	<0.001		3^rd^	5^th^	−3.57	0.336
	3^rd^	9^th^	−2.33	<0.001		3^rd^	9^th^	−7.10	0.008
Comparison point	1^st^	4^th^	0	N/A	Comparison point	1^st^	4^th^	0	N/A
(Acetone)H^+^ 59.04914	1^st^	8^th^	−0.27	<0.001	pET− CO_2_ [kPa]	1^st^	8^th^	−0.73	0.203
	1^st^	12^th^	0.25	0.086		1^st^	12^th^	−2.41	<0.001
	2^nd^	Max	0.02	0.238		2^nd^	Max	6.08	<0.001
	2^nd^	Forced	4.45	<0.001		2^nd^	Forced	−18.28	<0.001
	2^nd^	4^th^	1.41	<0.001		2^nd^	4^th^	−24.80	<0.001
	2^nd^	8^th^	1.32	0.001		2^nd^	8^th^	−7.91	<0.001
	3^rd^	1^st^	1.11	0.009		3^rd^	1^st^	−9.20	<0.001
	3^rd^	5^th^	0.54	0.053		3^rd^	5^th^	−10.82	<0.001
	3^rd^	9^th^	0.76	0.418		3^rd^	9^th^	−11.53	<0.001
B									
Protonated VOCs Mass [gm mol^−1^]	Minute	Breath	(%) Change of normalized mean values	Significance/Breath (P-value)	Hemodynamic parameters	Minute	Time [s]	(%)Change of normalized mean values	Significance/Time (P-value)
Comparison point	1^st^	4^th^	0	N/A	Comparison point	1^st^	40^th^ s	0	N/A
(Acetonitrile)H^+^42.03382	1^st^	8^th^	−3.34	0.125	Stroke volume [ml/beat]				
	1^st^	12^th^	−2.16	0.001		1^st^	60s	−0.27	0.819
	2^nd^	Max	1.71	<0.001		2^nd^	20s	−14.17	<0.001
	2^nd^	Forced	−0.26	0.001		2^nd^	40s	3.74	<0.001
	2^nd^	4^th^	−4.73	0.257		2^nd^	60s	2.12	<0.001
	2^nd^	8^th^	−2.46	0.048		3^rd^	20s	−1.04	0.014
	3^rd^	1^st^	−2.94	0.005		3^rd^	40s	−1.43	0.003
	3^rd^	5^th^	−4.94	0.008		3^rd^	60s	−2.39	<0.001
	3^rd^	9^th^	−5.64	0.007					
Comparison point	1^st^	4^th^	0	N/A	Comparison point	1^st^	40^th^ s	0	N/A
(Furan)H^+^ 69.03349	1^st^	8^th^	7.11	<0.001	Cardiac output [L/min]				
	1^st^	12^th^	3.43	0.001		1^st^	60s	0.14	<0.001
	2^nd^	Max	11.83	<0.001		2^nd^	20s	−6.45	<0.001
	2^nd^	Forced	−16.27	<0.001		2^nd^	40s	14.49	<0.001
	2^nd^	4^th^	−32.20	<0.001		2^nd^	60s	1.80	0.001
	2^nd^	8^th^	13.43	<0.001		3^rd^	20s	−1.22	0.120
	3^rd^	1^st^	4.68	<0.001		3^rd^	40s	−0.99	0.040
	3^rd^	5^th^	−3.25	<0.001		3^rd^	60s	−1.58	0.127
	3^rd^	9^th^	−5.43	<0.001					
Comparison point	1^st^	4^th^	0	N/A	Comparison point	1^st^	40^th^ s	0	N/A
(C_4_H_8_S)H^+^ 89.04195	1^st^	8^th^	−1.38	1.000	Pulse rate [beats/min]				
	1^st^	12^th^	−1.59	0.253		1^st^	60s	0.21	0.794
	2^nd^	Max	0.09	0.002		2^nd^	20s	12.04	<0.001
	2^nd^	Forced	−11.61	<0.001		2^nd^	40s	10.70	<0.001
	2^nd^	4^th^	−10.40	<0.001		2^nd^	60s	−0.74	0.018
	2^nd^	8^th^	−2.92	0.005		3^rd^	20s	−0.40	0.819
	3^rd^	1^st^	−3.64	0.039		3^rd^	40s	0.40	0.038
	3^rd^	5^th^	−8.14	<0.001		3^rd^	60s	0.88	0.006
	3^rd^	9^th^	−8.12	<0.001					

VOC concentrations from every forth exhalation as well as from the maximum and forced expiration of the second minute were compared to the 4^th^ breath of the first minute. Respiratory parameters were compared in the same way. (%) *Change of normalized mean values*: Positive values represent an increase and negative values represent a decrease. *Significance/Breath*: differences between the reference value and actual values were assessed by means of repeated measurement-ANOVA on ranks. Hemodynamic parameters were compared with the 40^th^ s of the first minute. *Significance/Time*: differences between the reference value and actual values were assessed by means of repeated measurement-ANOVA on ranks.

**Table 2:** Overall results of polynomial regressions.

**Order**	**R**^**2**^	**F-test**	**P-value**
1^st^	0.797	86.477	<0.001
2^nd^	0.855	62.032	<0.001
3^rd^	0.856	39.504	<0.001

The strengths of correlations between models and response variables are represented as R^2^ values. F-test simultaneously accesses multiple coefficients of regression and compares the fits of different linear models. The P-values stand for the statistical significance of corresponding F-test.

**Table 3:** Demographic data of healthy volunteers.

**ID**	**Age (years)**	**Sex**	**Height [cm]**	**Weight [kg]**	**Smoker**	**BMI**	**ID**	**Age (years)**	**Sex**	**Height [cm]**	**Weight [kg]**	**Smoker**	**BMI**
1	30	M	168	64	No	23	26	32	M	172	69	No	23
2	32	M	184	72	No	21	27	29	F	169	78	Yes	27
3	29	M	186	80	No	23	28	32	M	166	65	No	24
4	26	M	183	72	No	22	29	29	M	166	62	No	22
5	28	M	168	72	No	26	30	28	F	173	68	No	23
6	26	F	165	72	No	26	31	33	F	170	68	No	24
7	29	M	177	70	Yes	22	32	28	F	170	66	No	23
8	28	M	193	85	No	23	33	25	M	167	65	No	23
9	26	F	170	63	No	22	34	31	M	171	68	No	23
10	25	F	162	50	No	19	35	28	F	167	62	No	22
11	28	F	171	90	No	30	36	27	M	162	79	No	30
12	46	M	186	75	No	22	37	25	F	158	49	No	20
13	47	M	195	103	No	27	38	23	M	185	65	No	19
14	22	F	170	66	No	23	39	34	M	180	90	No	28
15	31	M	162	63	No	24	40	24	F	172	65	No	22
16	31	M	169	70	No	24	41	29	M	164	67	No	25
17	26	F	181	68	No	21	42	24	M	163	62	No	23
18	43	F	168	60	Yes	21	43	30	M	190	91	Yes	25
19	54	M	189	106	No	30	44	29	F	169	65	No	23
20	34	M	172	75	No	25	45	31	F	172	71	No	24
21	20	M	188	83	No	24	46	22	F	178	70	No	22
22	26	M	169	68	No	24	47	27	F	170	68	Yes	24
23	31	F	172	68	No	23	48	46	F	165	62	No	23
24	23	F	168	68	No	24	49	27	F	168	70	Yes	25
25	21	F	180	68	No	21	50	32	M	170	67	No	23

Data regarding participants’ age, sex, height, body weight, smoking habit and body mass index (BMI) are presented.

**Table 4:** PTR-ToF-MS-8000 measurement parameters for our study.

**Transfer line**		**Ion source**		**PTR drift tube**				**ToF-MS**
*Inlet Flow*	*Temp.*	*Inlet Flow*	*Current*	*E/N Ratio*	*Temp.*	*Pressure*	*Voltage*	*Mass resolution*
20 sccm	75 °C	6–7 sccm	4–5 mA	137–139 Td	75 °C	2.3 mbar	610 V	4000–5000 FWHM

Optimised operating values for transfer line, ion source, PTR drift tube and time of flight-mass spectrometry (ToF-MS) are presented.
