# Supplementary material for: FEV manoeuvre induced changes in breath VOC compositions: an unconventional view on lung function tests
Source: Sci Rep. 2016 Jun 17;6:28029. doi: 10.1038/srep28029 (PMC4911606; doi:10.1038/srep28029)
Supplement: Supplementary Information [file srep28029-s1.pdf]

FEV manoeuvre induced real-time changes in breath VOC compositions: an unconventional view on lung function tests

Pritam Sukul[1], Jochen K Schubert[1], Peter Oertel[1], Svend Kamysek[1], Khushman Taunk[2], Phillip Trefz[1], and Wolfram Miekisch[1,*]

[1]Rostock Medical Breath Research Analytics and Technologies (ROMBAT), Department of Anaesthesiology and Intensive Care, University Medicine Rostock, Schillingallee 35, Rostock, 18057, Germany
[2]Proteomics Lab, National Centre for Cell Science (NCCS), Pune University, Pune (Maharashtra), 411007, India
*corresponding author: wolfram.miekisch@uni-rostock.de

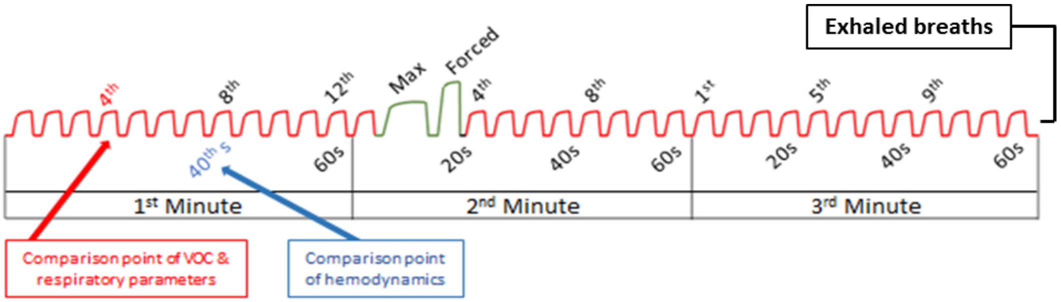

Supplementary Figure 1: Schematic overview of data points used for statistical comparison of differences. The 4th breath (in 1st min) represents such comparison point for VOC concentrations and respiratory parameters. Data from 40th s (in 1st min) was used for normalisation of hemodynamic parameters.

A.

			Exhaled VOC concentrations																	
			Normalized mean									Standard deviations								
	Breath	Time [s]	(Isoprene)H+	(Furan)H+	(Acetonitrile)	(Acetone)H+	(C2H6S)H+	(C4H8S)H+	(H2S)H+	(C6H6)H+	(C7H8)H+	(Isoprene)H+	(Furan)H+	(Acetonitrile)	(Acetone)H+	(C2H6S)H+	(C4H8S)H+	(H2S)H+	(C6H6)H+	(C7H8)H+
1st Minute	1	5	1.002	0.981	0.986	1.001	0.999	1.000	1.079	1.001	1.001	0.086	0.167	0.083	0.022	0.109	0.119	0.359	0.264	0.181
	2	10	1.011	0.980	0.987	1.001	1.003	0.996	1.075	0.971	0.986	0.107	0.168	0.081	0.022	0.096	0.111	0.374	0.244	0.182
	3	15	1.000	1.000	1.000	1.000	1.000	1.000	1.000	1.000	1.000	0.000	0.000	0.000	0.000	0.000	0.000	0.000	0.000	0.000
	4	20	1.003	0.981	0.985	1.000	1.014	0.996	1.096	1.000	0.999	0.087	0.169	0.084	0.018	0.099	0.106	0.476	0.265	0.178
	5	25	1.011	1.046	0.987	1.000	1.022	0.988	1.079	0.971	0.978	0.106	0.187	0.080	0.020	0.073	0.110	0.359	0.243	0.185
	6	30	1.030	1.029	0.982	0.999	1.019	0.985	1.100	0.928	0.943	0.111	0.176	0.084	0.024	0.083	0.113	0.334	0.257	0.186
	7	35	1.042	1.057	0.976	0.996	1.018	0.993	1.130	0.940	0.914	0.130	0.190	0.095	0.025	0.089	0.115	0.362	0.273	0.141
	8	40	1.061	1.061	0.967	0.997	1.013	0.986	1.112	0.892	0.896	0.146	0.206	0.064	0.019	0.080	0.113	0.423	0.214	0.144
	9	45	1.083	1.075	0.984	0.997	1.016	0.999	1.124	0.919	0.896	0.161	0.230	0.085	0.022	0.073	0.109	0.402	0.244	0.164
	10	50	1.077	1.077	0.986	1.001	1.013	0.995	1.164	0.988	0.900	0.191	0.217	0.088	0.022	0.089	0.115	0.467	0.241	0.165
	11	54	1.091	1.086	0.995	0.997	1.007	0.997	1.069	0.966	0.891	0.199	0.287	0.087	0.023	0.079	0.113	0.455	0.280	0.169
	12	59	1.111	1.074	0.978	1.003	1.017	0.984	1.067	0.965	0.895	0.218	0.228	0.097	0.031	0.098	0.127	0.477	0.307	0.163
2nd Minute	13	64	1.095	1.062	0.965	1.004	1.036	0.947	1.150	0.882	0.903	0.250	0.306	0.081	0.034	0.088	0.132	0.655	0.235	0.186
	M_{out}	70	1.212	1.128	1.017	1.000	1.070	1.001	0.955	0.884	0.903	0.281	0.278	0.101	0.031	0.074	0.160	0.404	0.249	0.131
	F_{out}	77	0.816	0.837	0.997	1.044	0.942	0.884	1.037	0.853	0.840	0.220	0.258	0.110	0.038	0.101	0.159	0.427	0.210	0.131
	16	84	0.627	0.678	0.953	1.014	0.872	0.896	1.013	0.890	0.895	0.179	0.249	0.107	0.027	0.114	0.148	0.449	0.227	0.171
	17	89	0.944	0.901	0.947	1.009	1.009	0.973	0.929	0.877	0.883	0.265	0.336	0.088	0.034	0.111	0.139	0.351	0.252	0.186
	18	94	1.101	1.006	0.969	1.014	1.045	0.981	1.033	0.893	0.882	0.285	0.302	0.084	0.033	0.095	0.160	0.539	0.276	0.167
	19	99	1.179	1.103	0.990	1.012	1.064	0.980	1.115	0.926	0.895	0.294	0.345	0.092	0.030	0.096	0.146	0.658	0.238	0.158
	20	104	1.212	1.134	0.975	1.013	1.080	0.971	0.983	0.902	0.863	0.302	0.270	0.098	0.026	0.090	0.146	0.465	0.227	0.152
	21	108	1.203	1.087	0.985	1.011	1.037	0.963	1.060	0.899	0.867	0.280	0.328	0.086	0.029	0.090	0.155	0.504	0.233	0.165
	22	113	1.165	1.083	0.972	1.008	1.038	0.978	1.032	0.882	0.868	0.264	0.263	0.103	0.035	0.087	0.152	0.435	0.211	0.156
	23	119	1.148	1.089	0.969	1.009	1.025	0.962	1.077	0.934	0.875	0.273	0.313	0.090	0.035	0.100	0.156	0.499	0.301	0.182
3rd Minute	24	124	1.123	1.047	0.971	1.011	1.039	0.964	1.029	0.886	0.864	0.264	0.247	0.085	0.034	0.089	0.142	0.421	0.218	0.149
	25	129	1.090	1.000	0.971	1.011	1.019	0.959	1.098	0.867	0.877	0.251	0.252	0.078	0.034	0.106	0.133	0.489	0.239	0.186
	26	134	1.071	1.003	0.960	1.005	1.004	0.947	1.148	0.909	0.853	0.238	0.271	0.078	0.032	0.088	0.149	0.605	0.285	0.154
	27	139	1.048	1.011	0.950	1.009	1.003	0.931	1.108	0.883	0.839	0.227	0.251	0.084	0.035	0.087	0.135	0.593	0.262	0.180
	28	144	1.016	0.967	0.951	1.005	0.985	0.919	1.058	0.867	0.863	0.233	0.244	0.085	0.033	0.099	0.153	0.561	0.230	0.193
	29	149	1.001	0.964	0.943	1.007	0.994	0.933	0.973	0.898	0.836	0.223	0.278	0.079	0.030	0.103	0.165	0.463	0.244	0.169
	30	154	0.985	0.937	0.944	1.009	0.994	0.922	0.999	0.881	0.823	0.213	0.231	0.085	0.031	0.094	0.153	0.380	0.280	0.162
	31	159	0.960	0.940	0.933	1.008	0.988	0.928	0.972	0.902	0.847	0.215	0.251	0.079	0.032	0.099	0.155	0.457	0.314	0.177
	32	164	0.950	0.946	0.944	1.008	0.977	0.919	1.031	0.894	0.832	0.207	0.309	0.084	0.033	0.084	0.166	0.472	0.278	0.147
	33	169	0.930	0.891	0.925	1.008	0.986	0.919	0.946	0.866	0.803	0.206	0.224	0.084	0.035	0.093	0.155	0.546	0.256	0.148
	34	173	0.945	0.892	0.904	1.008	0.969	0.904	0.980	0.839	0.791	0.218	0.195	0.098	0.032	0.090	0.146	0.488	0.263	0.153
	35	177	0.944	0.879	0.905	1.009	0.962	0.904	0.978	0.838	0.781	0.219	0.194	0.097	0.036	0.136	0.082	0.486	0.264	0.097

B.

			Respiratory parameters													
			Normalized mean							Standard deviations						
	Breath	Time [s]	Expiratory Tidal Volume (VTex)	pET-CO2	Minute ventilation (V'E)	RER	FIO2	V'O2	V'CO2	Expiratory Tidal Volume (VTex)	pET-CO2	Minute ventilation (V'E)	RER	FIO2	V'O2	V'CO2
1st Minute	1	5	0.954	0.998	0.970	0.893	1.003	0.926	0.958	0.149	0.037	0.149	0.086	0.005	0.157	0.151
	2	10	0.946	0.999	0.944	0.942	1.001	0.883	0.931	0.203	0.036	0.171	0.065	0.004	0.195	0.190
	3	15	1.000	1.000	1.000	1.000	1.000	1.000	1.000	0.000	0.000	0.000	0.000	0.000	0.000	0.000
	4	20	0.955	0.994	0.971	1.037	1.000	0.926	0.958	0.148	0.030	0.150	0.044	0.004	0.157	0.151
	5	25	0.945	0.995	0.943	1.059	1.000	0.883	0.931	0.202	0.038	0.170	0.067	0.004	0.195	0.190
	6	30	0.908	0.989	0.934	1.075	1.000	0.847	0.907	0.206	0.044	0.203	0.083	0.005	0.198	0.208
	7	35	0.902	0.991	0.926	1.085	1.000	0.854	0.923	0.205	0.053	0.217	0.104	0.004	0.168	0.182
	8	40	0.914	0.993	0.943	1.095	0.999	0.841	0.914	0.195	0.058	0.214	0.123	0.005	0.164	0.172
	9	45	0.963	0.988	0.960	1.110	0.999	0.853	0.937	0.206	0.062	0.213	0.141	0.005	0.167	0.168
	10	50	0.928	0.977	0.959	1.121	0.999	0.829	0.919	0.194	0.063	0.207	0.154	0.005	0.179	0.184
	11	54	0.901	0.978	0.929	1.132	1.000	0.818	0.914	0.227	0.067	0.222	0.168	0.004	0.208	0.211
	12	59	0.920	0.976	0.931	1.136	1.000	0.787	0.882	0.186	0.072	0.219	0.172	0.005	0.185	0.189
2nd Minute	13	64	0.910	0.967	0.911	1.130	1.000	0.872	0.967	0.224	0.067	0.214	0.178	0.005	0.279	0.281
	M_{out}	70	2.063	1.061	1.414	1.158	0.999	1.339	1.487	0.853	0.080	0.445	0.220	0.005	0.640	0.587
	F_{out}	77	3.917	0.817	2.714	1.469	0.997	1.292	1.879	1.787	0.072	0.990	0.257	0.008	0.546	0.757
	16	84	2.650	0.752	2.458	1.805	1.002	0.875	1.638	1.104	0.085	1.242	0.337	0.005	0.442	0.950
	17	89	1.346	0.863	1.364	1.683	1.002	0.711	1.176	0.465	0.077	0.417	0.406	0.006	0.243	0.409
	18	94	1.100	0.897	1.162	1.515	1.002	0.708	1.048	0.359	0.071	0.383	0.372	0.005	0.219	0.343
	19	99	1.092	0.921	1.157	1.373	1.001	0.800	1.069	0.285	0.066	0.302	0.312	0.005	0.211	0.262
	20	104	1.073	0.921	1.112	1.309	1.001	0.804	1.026	0.327	0.070	0.325	0.288	0.005	0.230	0.267
	21	108	1.063	0.917	1.076	1.282	1.000	0.780	0.977	0.273	0.069	0.268	0.280	0.006	0.176	0.209
	22	113	1.057	0.914	1.079	1.265	1.000	0.776	0.961	0.306	0.076	0.327	0.274	0.006	0.221	0.268
	23	119	1.010	0.906	1.020	1.255	1.000	0.751	0.925	0.294	0.079	0.288	0.271	0.005	0.175	0.224
3rd Minute	24	124	0.999	0.908	0.998	1.239	1.000	0.752	0.910	0.312	0.082	0.305	0.284	0.006	0.236	0.271
	25	129	0.932	0.903	0.965	1.218	1.000	0.733	0.872	0.255	0.079	0.278	0.273	0.005	0.192	0.234
	26	134	0.978	0.899	0.995	1.204	1.000	0.741	0.876	0.342	0.079	0.316	0.268	0.005	0.222	0.277
	27	139	0.952	0.897	0.970	1.193	0.999	0.724	0.851	0.310	0.079	0.310	0.270	0.006	0.190	0.245
	28	144	0.953	0.892	0.964	1.188	0.999	0.729	0.842	0.312	0.087	0.302	0.276	0.006	0.215	0.261
	29	149	0.927	0.891	0.925	1.176	1.000	0.726	0.835	0.331	0.087	0.337	0.264	0.005	0.233	0.300
	30	154	0.913	0.890	0.925	1.162	0.999	0.719	0.820	0.308	0.086	0.283	0.255	0.007	0.200	0.235
	31	159	0.933	0.887	0.934	1.155	1.000	0.722	0.824	0.297	0.089	0.269	0.247	0.005	0.158	0.216
	32	164	0.924	0.885	0.929	1.158	1.000	0.714	0.812	0.283	0.086	0.269	0.245	0.005	0.194	0.235
	33	169	0.935	0.881	0.917	1.151	1.000	0.735	0.829	0.280	0.086	0.267	0.244	0.005	0.200	0.216
	34	173	0.924	0.884	0.928	1.133	1.000	0.727	0.808	0.282	0.089	0.270	0.235	0.004	0.204	0.220
	35	177	0.936	0.885	0.918	1.146	1.000	0.727	0.808	0.281	0.088	0.266	0.251	0.005	0.204	0.220

C

		Hemodynamic parameters							
		Normalized mean				Standard deviations			
	Time [s]	Cardiac Output	Stroke Volume	Pulse Rate	MAP	Cardiac Output	Stroke Volume	Pulse Rate	MAP
**1st Minute**	20	0.980	0.982	1.003	0.999	0.038	0.056	0.029	0.052
	40	1.000	1.000	1.000	1.000	0.000	0.000	0.000	0.000
	60	1.009	0.997	1.000	1.023	0.041	0.042	0.045	0.047
**2nd Minute**	80	0.949	0.882	1.120	1.046	0.062	0.074	0.084	0.090
	100	1.145	1.037	1.107	0.984	0.077	0.074	0.080	0.079
	120	1.018	1.021	0.993	1.039	0.072	0.066	0.076	0.084
**3rd Minute**	140	0.988	0.990	0.996	1.039	0.072	0.057	0.069	0.081
	160	0.990	0.986	1.004	1.032	0.071	0.054	0.077	0.072
	180	0.984	0.976	1.009	1.025	0.072	0.053	0.079	0.069

Supplementary Table 1: **Normalized mean values and variations of different VOC concentrations, respiratory and hemodynamic parameters.** In section **(A)** and **(B)**, maximum exhalation and forced expiration are marked respectively as $\mathbf{M_{out}}$ and $\mathbf{F_{out}}$. **(A)** Normalized (normalized on 3rd breath) means (from 50 volunteers) and standard deviations of 9 different VOC concentrations in each breath during the measurement. **(B)** Normalized (normalized on 3rd breath) mean (from 50 volunteers) and standard deviations of 7 different respiratory parameters in each breath during the measurement. **(C)** Normalized (normalized on 40th s data point of the 1st min) means (from 50 volunteers) and standard deviations of 4 different hemodynamic parameters during the measurement are presented.
